# Spinal Concussion

**Published:** 1894-12

**Authors:** James Johnston

**Affiliations:** Houston; Brownwood, Texas


For Texas Medical Journal.

### SPINAL CONCUSSION.

BY JAMES JOHNSTON, M. D., BROWNWOOD, TEXAS.

CONCUSSION of the spine, railway spine, concussion of the spinal cord, traumatic psychosis, hypnosis, traumatic neurosis, meningomyelitis, railway brain, hysteria, paralysis, hyperæsthesia, anesthesia, hypnotism, melancholia, hypochondriassis, hypnosis, traumatic hysteria, traumatized mind, psychoneurosis, hysterical and neurasthenical conditions, lumbar cervico dorsal sprain, sprain of the whole vertebral column, sacral sprain, general nervous shock, functional or dynamic disturbance of the nervous equilibrium, nervousness, photophobia, asthenopia, parasthesis, neurasthenia, the sensory ganglia, the medulla oblongata, stomato-gastric respiratory ganglia, diversity of auhoritative medical opinion, its multitudinous symptomatology and its psychology, with sundry other pathognomonic conditions (connected with railroad spine) too numerous to mention.

Above you have a few of the modern technical terms used in this discussion on spinal shock. Oh! what a long tail our cat has got! Is it any wonder that a jury of farmers give heavy damages against a railroad company after hearing testimony involving such terrible terms?

For some time, I have been reading the discussions started in the TEXAS MEDICAL JOURNAL, and now going the round of the medical press, on the above subject, with some interest, a great deal of disgust, and more mortification. I am now on the shady side of fifty, and have studied medicine since I was a boy. I have been in active practice for thirty years, have made the nervous system a *special study* for the last twenty-five years, and, with all of this, I know but little on the subject, concussion of the spine; and the more I hear the arguments for and against it, the more cloudy the subject appears to me.

The discussion of this subject reminds me of the Scotchman's definition of politics, viz.: "When a man is talking about what he (kens) knows nothing about, and the man he is talking to does not understand him." That, he says, is talking politics.

The reason this subject does not appear clear to me may be for lack of higher education in my youth. The only letters after my name are M. D. I have never been a "professor" in a college, neither am I a Latin scholar. I know a few Latin phrases, but I am not so vain or foolish as to put them in print for school children to laugh at, as some writers do.

Pope says, "A little learning is a dangerous thing." Some men get a run through a literary institution, and come out with as many letters after their names as there are knots to the tail of a kite; then they profess themselves authority on all subjects, and think that all the world is looking up to them—like Goldsmith's Village Schoolmaster, "And still the wonder grew that one small head could carry all he knew." If one of them happens to declare the horse is sixteen feet high, he is prepared to prove it, and if the world should refuse to agree with him, he, like the lone juryman who often stands out against the other eleven, thinks they are all fools.

It is evident to every disinterested person that this discussion was not gotten up for the benefit of science. It appears to tend toward doing away with certain principles or conditions in surgery that have been operating seriously in the way of excessive and wrongful damages against railroad companies, etc.

I am not interested on either side of the discussion, and I have no personal feelings whatever for or against any of those who are engaged in it; I do not know one of them personally; but I am jealous for the medical profession (that we have lately heard so much about, as being "tough," etc.), as well as for right and justice.

Railroads are a blessing to the world, and especially to Texas. In law, their rights are the same as individuals.

I have been called into court several times to give testimony, and sometimes it has been for, and sometimes against railroad companies. If properly represented, I have always found them willing and ready to pay all reasonable damages for injuries sustained.

There are some who believe that railroad companies are monsters for robbing the people. Here is a railroad train going at a speed of thirty, forty, or sixty miles an hour. Think of it; it is a sight,—a most terrible thing. If an old Texas long-horn cow happens to be on the track, too poor to get out of the way, in a second she is knocked into a full blooded Jersey or a Durham, with a pedigree as far back as the Irishman's, who said, "When Noah was saved in the Ark, one of me ancistry saved himself in a boat of his own kinstruction." Instead of the railroad company having to pay $5, the real value of the long-horn, it is compelled to pay $100, the price of a Jersey or Durham. It is known, almost to every one, that railroad companies have to pay excessive damages, sometimes wrongfully, because the science of medicine and surgery has not reached that point where the court and jurors of our country can get clear, reliable, intelligent testimony sufficient to enable them to render a just verdict. In all cases, and as in criminal cases, under the law, when a doubt exists, the rule in practice is to give the defendant the benefit of it. So it is in civil suits against railroad companies; the rule is to give the claimant the benefit of the doubt. To remedy this, some lawyers, and some doctors, drawing large salaries from railroad companies, are moved with a desire to render the best possible service to their employers, or else for some cheap notoriety, have agreed upon a plan to remedy this evil. They perceive the cause of this trouble to be smart attorneys and Erichsen's little work on "Concussion of the Spine," and seeing no way to get rid of the former, their whole force is let loose against the latter, and a few of the great lights (?) have turned loose upon the little book of the author,—one of the greatest surgeons the world has ever yet seen, denouncing him and his work in strong epithets, such as "vampire," etc.; and in order to do their work more effectually, they do not confine themselves to the use of medical journals, "the proper organs for the discussion of medical science," but have their views published in the *Galveston News*, and other lay publications. But when they thought that they had succeeded in killing their most formidable enemy, lo and behold! a David steps forth, with his little pebble and his little sling, and takes a crack at the hydraheaded Goliath!---Swearingen's criticism appears. It comes out in that champion journal of the cause of science and of the dignity of legitimate medicine, the fearless "Red-Back." It appeared, like Banquo's ghost, to trouble them, and would not "down."

Then our learned friend, Dr. W. B. Outten, of St. Louis, gets off a long and labored article to prove that Dr. Swearingen's theory is wrong, because, he says, he cannot prove it by post-mortem examination, when any tyro in medicine knows that no post-mortem examination ever conducted by the most skillful hand, and the use of the most powerful microscope, has ever shown alteration of the nerves sufficient to account for neuralgia, or other diseases of the nervous system, apart from nervous shock.

In the course of his arguments, Dr. Outten says, in substance, that traumatic neurasthenia can be produced by hypnotism, etc., and then winds up by saying that he is honest, and that he has stated facts(?) for Swearingen to disprove.

Now, I would like to know the mode of investigation the gentleman has adopted that enables him to state so positively that his theory is true. Was it from post-mortem examination, or from the array of authorities he has quoted?

Our learned friend from Waco then follows, in somewhat the same strain, but is more modest and not so positive. He thanks Dr. Swearingen for "compliments," uses considerable irrelevant argument to prove nothing in particular, and deprecatingly mentions how hasty the first article was written; "written with pencil," etc. A reader of his rejoinder would naturally conclude that the doctor felt as if he had been decoyed into bad company, and that now he really wishes he had not done it. This is very commendable. Great and wise men sometimes change their views, but fools never.

Those advocates so eager to do away with railroad spine have defeated their own object by establishing the fact that traumatic neurasthenia (the same thing) can be manufactured at will. All that is necessary is for a passenger train to stop suddenly and produce a slight jar. When this occurs near a large city, there will be lots of those "sharp attorneys" to go to work on the passengers, and tell them that their spines are badly shocked, and that they are injured for life, and at the same time give each one a copy of that iniquitous little book of Erichsen's on "Spinal Concussion." Then put the patient to bed, give him light diet, and keep Erichsen's little book at the bed side. Have it explained by the family doctor and the attorney. In this way it will take but a few days to produce a state of hypnosis, and we have a real case of "traumatic neurasthenia."

Now, if this is true, and we cannot doubt it (because these great latter-day lights say so), railroad suits will multiply without limit or cessation, for damages done to the nervous system. Instead of millions being wrongfully extorted from railroad companies in the past, billions will be in the future, because these great lights, in their very honest and innocent efforts to break down the evil "Erichsen and his little book" have blundered, exposed the trick, and given the whole snap away to the smart attorneys:—only, they call it by another name.

This exposure will not only affect the railroad practice, but it will also now take its course in general practice,—of both law and medicine. If a case can be produced by hypnotism, cases can be cured by the same means, eh?

This will be a boon to suffering humanity, as no medicine will be needed, and no drug bills to pay. Outten, Outten, what have you done!

The magic wand will take the place of the pill bags, thousands of long-haired, lean faith-doctors will get fat, Christian Science will be revived, and scores of other "sciences" will spring into existence. But the poor railroad companies,—what will become of them? This great, huge anaconda will open its mouth like a mighty chasm, and swallow them up, so that there will not be a remnant of them left for the government to buy up, when the third party gets into power.

This—laying all jokes aside—is a great and terrible evil brought about by a few overzealous employes, who do not fairly represent the railroad companies, nor the band of noble, honest railroad surgeons of our country, and for which the railroad companies and railroad company surgeons, as a class, should not be held responsible. We can always find some of these self-wise and great men with numberless letters after their names, indicating title, honor, etc., with much learning and less common sense, in all classes and associations; and instead of being an advantage to society, they are really a great disadvantage, because they are always either in trouble themselves, or are getting others into trouble.

The fact that heavy damages are sometimes obtained from railroad companies, is no reason why these late authorities should try to do away with certain principles or conditions in surgery which are well established. They might just as well say that because they have to pay $100 00 for a $5.00 long-horn cow, represented as a fine Jersey or Durham, that there is no such thing as a Jersey or a Durham cow in existence.

I stated at the outset that I felt mortified on reading this discussion, It is disgraceful. I am sorry that such a thing should have occurred in the United States, and especially in Texas.

The arguments made against spinal concussion are misleading, and can not be in the interest of truth and science. They are contrary to the authorized opinions of ninety-nine per cent. of all the best and most reliably educated physicians throughout the entire civilized world.

I must say in justice to the Texas medical journals, that they have treated this subject fairly and ably, and would especially commend the editorial in the TEXAS MEDICAL JOURNAL for September.

In conclusion, I want to notice, briefly, Hon. Clark Bell's remarks on Swearingen's criticisms, asking him to withdraw them, etc. This, I think, is about the most impudent, out of place, and unbecoming the editor of a medico-legal journal, of anything I have ever read.

I have not entered into these remarks on this subject from a scientific standpoint; but if I have succeeded in showing it up to be so ridiculous that it will be stopped, and future discussions of its kind not in the interest of science, I will feel that I have in a measure succeeded in accomplishing my object, and science may be benefited indirectly.

---

For Texas Medical Journal.

